# Brain Waves Evaluation of Sound Therapy in Chronic Subjective Tinnitus Cases Using Wavelet Decomposition

**DOI:** 10.3389/fnint.2018.00038

**Published:** 2018-09-19

**Authors:** Abdoreza Asadpour, Mehran Jahed, Saeid Mahmoudian

**Affiliations:** ^1^Department of Electrical Engineering, Sharif University of Technology, Tehran, Iran; ^2^ENT and Head & Neck Research Center, Iran University of Medical Sciences (IUMS), Tehran, Iran

**Keywords:** tinnitus, Electroencephalogram, Residual inhibition, visual analog scale, wavelet decomposition

## Abstract

Management and treatment of subjective tinnitus is an ongoing focus of research activities. One of the most viable assessments of such treatment is the evaluation of brain activity in addition to patient response and clinical assessment. This study focuses on sound therapy and evaluation of patients’ electroencephalogram (EEG) in order to verify the potency of this approach. Broadband sound therapy was applied to nineteen participants aging from 25 to 64 and suffering from chronic subjective tinnitus to study the difference of brain activity, a) before fake treatment, b) after fake treatment and c) after the main treatment, using EEG and Visual Analog Scale (VAS) for evaluating Residual Inhibition (RI). Four features were extracted using 4-level wavelet decomposition with Symlet 8 as its mother wavelet. For the “After the main treatment” stage, the mean value of wavelet coefficients for the last wavelet level, which corresponded to delta band of EEG, was lower in the FC3 channel based on Two-Sample *T*-Test with significance level of 0.01, as compared to the same channel of the “before the treatment” stage, for cases in which decreased tinnitus loudness were reported.

## Introduction

Subjective tinnitus is defined as the perception of sound without an external sound source (Eggermont and Roberts, 2004; Shargorodsky et al., 2010). Subjective tinnitus can only be experienced by the subject, so the perception experience is reliant on self-reporting (Heller, 2003). Possible event onsets for tinnitus include age-related hearing loss, loud noise exposure, and ototoxic medications (Dan, 2005; Bauer and Brozoski, 2008; Shargorodsky et al., 2010; American Tinnitus Association, 2017). Chronic tinnitus is the lack of remission of tinnitus symptoms, and tinnitus perception lasting above 2 years is considered permanent (Han et al., 2009).

Though there is not yet a cure for tinnitus, there are several tinnitus management options that are based on current neuroscientific models and theories of tinnitus. Positive outcomes have been reported for treatment options, such as sound therapies, electrical stimulation, and pharmaceuticals (Tyler et al., 2007). Of these three main treatment options, electrical stimulation and pharmaceuticals are mainly experimental and are not routinely used with tinnitus patients. Sound therapy treatment options have included hearing aids, personal listening devices, sound generators, maskers, and music (Henry et al., 2009). Tinnitus masking uses noise generating devices. Tinnitus patients are instructed to play the noise at a level above the tinnitus perception thereby masking tinnitus and providing temporary relief (Henry et al., 2002). Measuring Residual Inhibition (RI) helps to further evaluate the characteristics of an individual’s tinnitus and illustrate that a broadband noise stimulus can positively change and affect a tinnitus perception (Henry et al., 2005). RI refers to the phenomenon whereby the tinnitus perception is reduced in intensity, or eliminated altogether, following auditory stimulation (Vernon and Meikle, 1988).

Brain activity in tinnitus cases has been investigated by several studies using Electroencephalogram (EEG) and Magnetoencephalogram (MEG). In one study, MEG activity was recorded from nine awake human controls and nine patients suffering from severe neurological or neuropsychiatric disorders, including one tinnitus patient. The averaged power spectra showed a decrease in alpha power and an increase in theta band and gamma band activities in patients in comparison to the control group (Llinás et al., 1999). In another study, neuromagnetic evoked fields of 14 tinnitus patients with high frequency hearing loss and 11 normal hearing controls were measured using lesion-edge or control (an octave below lesion-edge) tones, where responses to control tone were reported to be larger in patients in comparison to the control group (Weisz et al., 2005). In a more recent study, EEG recordings of eight subjects with intractable unilateral tinnitus were compared to 25 control subjects. Focal unilateral areas of gamma (>40–80 Hz) band activity (Hot spots) were found in the auditory cortex of the tinnitus subjects, but not in the control subjects (Ashton et al., 2007). Kahlbrock and Weisz studied eight chronic tinnitus patients including the patients with hearing loss in order to evaluate the effect of RI on their brain activity using tonal sounds as stimuli and reported a significant reduction of normalized power in the delta band after RI (Kahlbrock and Weisz, 2008). In another study, the effect of current delivery was evaluated through implanted electrodes at the Blood Oxygen Level-Dependent (BOLD) activation hotspots. The investigators reported increased gamma and theta bands activity and suggested that theta-gamma coupling might be related to tinnitus (De Ridder et al., 2011). Adjamain and colleagues utilized MEG signal to measure brain activity in four groups of participants, namely tinnitus with hearing loss, tinnitus with clinically normal hearing, no tinnitus with hearing loss, no tinnitus with clinically normal hearing in resting state, and during the presentation of 0.1–12 kHz masking noise. They observed higher delta band activity calculated based on mean power spectra in the “tinnitus with hearing loss” group as compared to the “no tinnitus with normal hearing” group, suggesting the necessity of combination of tinnitus and hearing loss for increased delta band activity. Their study further reported reduced delta band activity for 21 patients who reported RI and 13 others inclusive of three normal hearing subjects (Adjamian et al., 2012). The effect of RI and Residual Excitation (RE) were investigated in a group of 17 chronic tinnitus patients including eight patients with normal hearing and. Also, maskers with central frequency of 1.2–7 kHz, and bandwidths of 0.025–0.525 kHz were used to produce RI. They reported that 6 of the 14 patients who had reported RI, demonstrated a positive correlation between the tinnitus intensity and both delta and theta bands, while eight patients showed this positive correlation in gamma band based on mean power calculation (Sedley et al., 2012). In another research, resting state EEG recordings and behavioral data were used to study the emotional and cognitive state of 24 chronic tinnitus subjects and to compare them to 24 unaffected control subjects. Tinnitus perception intensity was marked by increased power in the delta, alpha, and lower gamma frequency bands, over both temporal lobes and over the left perisylvian area. Tinnitus distress intensity was marked by increased upper beta band activity over the frontal lobe area (Meyer et al., 2014). In another study by Sedley et. al., brain activity from a 50 years old awake behaving bilateral tinnitus subject was logged using extensive intracranial recordings while tinnitus loudness perception was modified through acoustic stimulation. They observed delta and theta power reduction in auditory cortex (Sedley et al., 2015). These findings show that different aspects of the tinnitus perception can be objectively measured by distinct changes in EEG activity. As previous studies did not evaluate the effect of broadband sound therapy on EEG brain waves in chronic tinnitus patients with normal hearing, this study intends to utilize appropriate experimental protocol and analyses in order to find new features to investigate possible effects. We start with a brief theoretical background of our proposed analytical approach. In methods section we introduce experimental design, acquisition protocol and proposed features. Finally results and conclusion will provide our findings and their justification.

## Theoretical Background

### Wavelet Decomposition

The application of wavelet-based analysis to neuronal waveforms such as EEG has been demonstrated to offer advantages in signal detection, component separation, and computational speed over traditional time and frequency techniques (Samar et al., 1999).

Wavelets are oscillating amplitude functions of time that must satisfy several conditions: a wavelet ψ is a function that is zero average over time and has unit energy. The amplitudes of a wavelet have large fluctuations within a designated time period and extremely small values outside of that time while being band-limited in terms of their frequency content. This property allows them to be localized in time and frequency (Samar et al., 1999). The wavelet chosen to perform a wavelet transformation is called the mother wavelet. During a wavelet transformation the signal of interest gets transformed into a representation that can demonstrate frequency content at different points in time. The concept is very similar to a windowed Fourier transform because it measures time-frequency variations of the spectrum, but has a different time-frequency resolution (Mallat, 1999). The Continuous Wavelet Transform (CWT) of a signal f can be calculated using following formula where ^∗^ indicates the complex conjugate. Varying the values for s and u results in an infinite number of combinations that can be used to decompose the signal, f (Mallat, 1999).

$$F(u,\;s)=\int_{-\infty }^{\infty }f(t)\frac{1}{\sqrt{s}}\text{ψ}*\left(\frac{t-u}{s}\right)dt$$

However, the CWT is very inefficient because of the redundancy that occurs when displaying closely spaced time points (Samar et al., 1999). A much more computationally efficient approach is the use of the Discrete Wavelet Transform (DWT) (Mallat, 1999). In DWT, The original signal is convolved with a low and high pass filter whose impulse response is determined by the wavelet chosen. The output of each filter produces the same number of samples as the original signal, so both outputs are down-sampled by two resulting in the approximation and detail coefficients each with half the number of points as the original signal. The coefficients represent a correlation between the signal of interest and wavelet chosen at different scales and during translation. Because all of the coefficients are preserved, the original signal or any level of decomposition can be reconstructed. To decompose the signal more than one level, the detail coefficients are stored and approximation coefficients are used as the signal and the process repeats.

## Materials and Methods

### Experimental Design

In this study, the effect of acoustic treatment was examined on subjective tinnitus cases given the state of their tinnitus was ongoing for at least 6 months. The acoustic treatment was applied in two phases: (1) Fake sound therapy (Placebo) in which a periodic 1 s sound wave of 500 to 1500 Hz frequency band and intensity of 20 dB in sound pressure level (SPL) and 4 s silent gap were applied to the participants for 1 min. (2) A broadband sound containing 2 to 10 kHz frequencies with the intensity of 70 dB SPL was presented for 1 min. All sounds were played through a stereo headphone in order to cause RI effectively. Before and after each phase, visual analog scale (VAS) was obtained from participants to measure the loudness of the participant’s tinnitus with a user interface software which presented a number scale ranging from 0 (“None”) to 10 (“Extreme”) while QEEG was recorded continuously during the whole procedure. VAS is an assessment to measure a characteristic -such as pain, loudness or annoyance- which has a range across a continuum values and cannot be measured directly. These scales are highly subjective therefore they are valuable when changes within individuals are measured (Gould et al., 2001).

### Subjects

Nineteen participants (13 male and 6 female subjects), ranging from 25 to 64 years, inclusive of subjective tinnitus individuals who suffered from the symptom for at least 6 months with normal hearing participated in the study. Pure-tone audiometry was conducted for frequencies between 0.25 and 8 kHz. Normal hearing was defined as thresholds ≤ 20 dB hearing level at standard audiometric frequencies from 0.25 to 8 kHz. All subjects reported that Persian was their first language and none of them wore hearing aids or had previously participated in a formal tinnitus treatment program.

### Acquisition Protocol

For the EEG setup, subjects had their eyes opened and were seated in an upright comfortable chair located in an electromagnetic shielded booth. A 32-channel recording EEG device was used in which 32 Ag/AgCl electrodes were situated according to the International 10–20 Standard System. Two channels were utilized to record Electro-oculogram, two other channels to record left and right mastoid potentials and one channel to acquire trigger signal. The electrodes were examined against the reference electrode (Tip of Nose) to keep the impedances under 10 kΩ using an impedance monitor which was built-in to the recording program. Subjects were asked to remain still, relax their face, and focus their eyes on one spot to minimize muscle artifacts. EEG signals were sampled at the rate of 1 kHz, and band-pass filtered at 0.4–200 Hz where a notch filter of 50 Hz was applied to remove the power supply noise.

**FIGURE 1:**
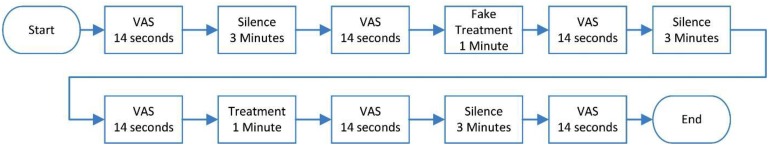
The sequence of data acquisition procedure.

The acquisition procedure was applied using the Presentation software (Neurobehavioral Systems) and began with answering VAS for 14 s and 3 min without presenting any sound. VAS was taken again for 14 s and then fake treatment was applied for 1 min and after the fake treatment, VAS was again obtained for 14 s, and 3 min without any sound followed by VAS for 14 s which was applied to the participants. Then main sound treatment was applied for 1 min and VAS was taken once more for 14 s and after that, the last 3 min of silence and VAS for 14 s were presented. During the whole procedure, EEG signals were recorded. **Figure 1** shows the sequence of this data acquisition procedure.

### Pre and Post Processing and Analysis

The recorded data were imported to MATLAB 2013a and filtered using an FIR band-pass filter ranging from 1 to 64 Hz. Eye-blinking and ECG artifacts were removed using ICA. ICA algorithm used in this study was Runica and removed components were selected manually by the user. The FIR filter and ICA were created by EEGLAB toolbox for MATLAB (Delorme and Makeig, 2004).

**FIGURE 2:**
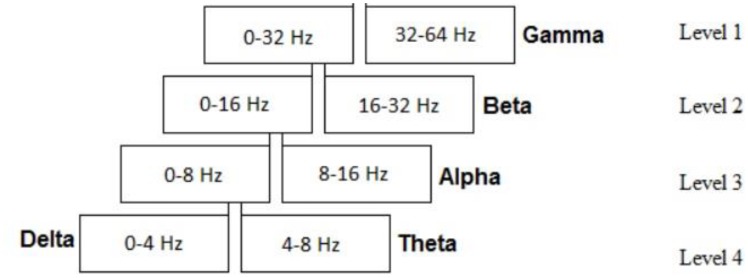
Decomposition wavelet levels and the corresponding EEG bands.

Discrete Wavelet Decomposition was used for time and frequency analysis and to extract EEG bands. Five EEG bands were decomposed, namely delta, theta, alpha, beta, and gamma. As the signal was filtered, four levels of decomposition were needed for extracting these bands. **Figure 2** shows the levels and EEG bands. For subsequent wavelet analysis of the EEG bands, Symlet 8 was used as the appropriate mother wavelet (Samar et al., 1999). Additionally, eight mother wavelets including Biorthogonal 3.5, 4.4, 5.5, Daubechies 5, 8, and Symlet 4, 5, 8 were tested using wavelet reconstruction on 50% of all the data and the Euclidean Distance from the original signal showed that best result was achieved through the Symlet mother wavelet. It is worth mentioning that the maximum average distance from the original signal for all above mother wavelets was lower than 3 × 10^-4^%.

An important approach utilized in this study was to choose features from the wavelet analysis in order to differentiate between signals with and without RI. Four features were calculated for each of the EEG bands of the participants, as noted below,

$$\begin{array}{l} \text{Feature 1}=\frac{1}{\text{n}}\sum_{\text{i}=1}^{\text{n}}C_{i}\\ \text{Feature 2}=\frac{1}{\text{n}}\sum_{\text{i}=1}^{\text{n}}|{\text{C}}_{\text{i}}{|}^{2}\\ \text{Feature 3}=\sqrt{\frac{1}{\text{n}}\sum_{\text{i}=1}^{\text{n}}{\left({\text{C}}_{\text{i}}-{\text{C}}_{\text{mean}}\right)}^{2}}\\ \text{Feature 4}=\frac{\frac{1}{\text{n}}\sum_{\text{i}=1}^{\text{n}}\left|{\text{C}}_{\text{i},\text{j}}\right|}{\frac{1}{\text{n}}\sum_{\text{i}=1}^{\text{n}}\left|{\text{C}}_{\text{i},\text{j}+1}\right|},\text{j}=1,2,3,4 \end{array}$$

where C_i_ is the wavelet coefficient for each band and j represents a particular band. Briefly, Feature 1 is the mean of each band coefficients while Feature 2 is the mean energy of each band that roughly represents the brain activity of a given EEG band. Feature 3 represents the standard deviation of coefficients in each band, and finally Feature 4 is the ratio between the mean absolute value of a given EEG band’s coefficients and the its immediate neighboring band.

## Results

**FIGURE 3:**
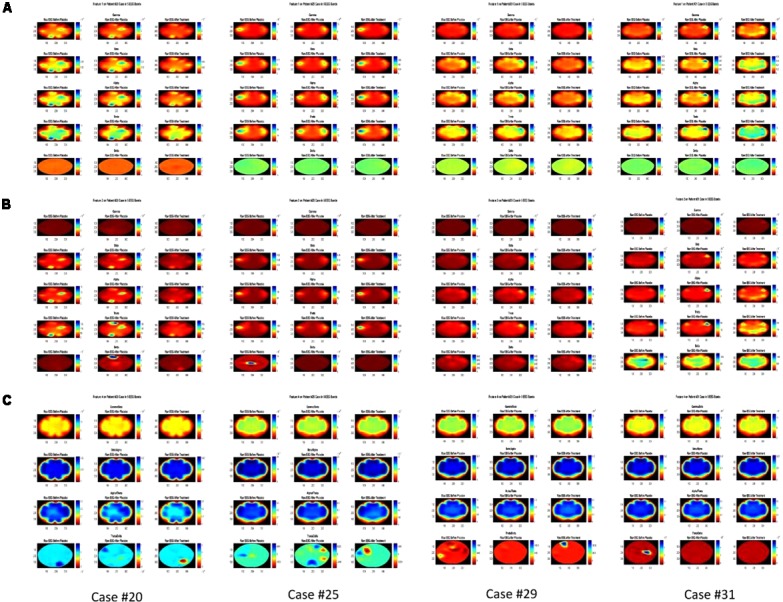
Brain map of the Features 1, 2, and 4 in three colums before fake treatment (Placebo), after fake treatment and after the main treatment for each Feature and each Case, for 4 participants (Case #31 showed no RI). **(A)** Feature 1 in Gamma, Beta, Alpha, Theta, and Delta band from top to bottom for each Case. **(B)** Feature 2 in Gamma, Beta, Alpha, Theta, and Delta band from top to bottom for each Case. **(C)** Feature 4 in Gamma/Beta, Beta/Alpha, Alpha/Theta, and Theta/Delta from top to bottom for each Case.

Three periods of signals for each case were selected for analysis and the features were extracted from these signals for comparison. Accordingly, these were three silent minutes EEG recording before the fake treatment, three silent minutes after the fake treatment, and three silent minutes after the main treatment. Signals were filtered and the artifacts were removed using ICA. Then a 4-level wavelet decomposition was applied to calculate the coefficients of each EEG band and the features were subsequently extracted. As an example for showing how the experiment changes the activity in different regions of the subjects’ brain, **Figure 3** represents the brain map of Features 1, 2, and 4 for four of the participants. The evaluation of VAS indicated that eleven cases had RI after the main treatment while other eight participants did not report any RI. It was observed that based on Feature 1, the activity due to tinnitus occurring in specific regions of the brain is different between these two groups. To extract the best EEG channel objectively, a two-sample *T*-test with varying significance level ranging from 0.01 to 0.25 was applied on the ratio of Feature 1, 2, and Feature 3 after the main treatment and before the fake treatment between the group with RI and the group without RI for all EEG channels. This ratio was used to normalize the features for each subject and each EEG channel of a group was compared to the same channel in the other group. Two Sample *T*-test indicated that at significance level of 0.01, channel FC3 showed meaningful difference between the two groups in delta band for Feature 1 (*p* < 0.004) while at significance level of 0.09, channel F8 had meaningful difference in delta band for Feature 2. The results show that after the occurrence of RI, the mean value of coefficients in delta band (Feature 1) decreased while for cases who did not show RI, it could not be seen. Feature 4 depicts that no significant differences between groups were observed.

**FIGURE 4:**
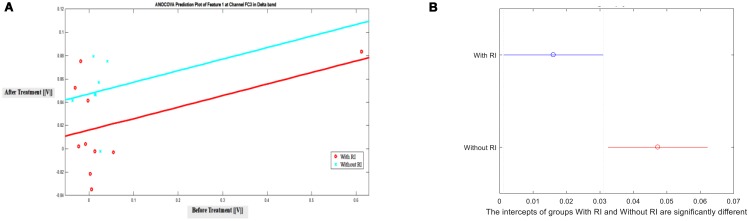
ANOCOVA of Feature 1 in channel FC3 between groups with and without RI in Delta Band at significance level of 0.05. **(A)** Prediction, **(B)** Comparison of intercepts.

Additionally, after observing the significant difference in *T*-Test, Analysis of Covariance (ANOCOVA) was applied to Feature 1 in channel FC3 with Parallel Lines method at significance level of 0.05 to verify the results of *T*-Test. It showed that the intercepts of the two groups are significantly different. **Figure 4** shows the lines and the comparison of ANOCOVA. It is worth mentioning that each band has its own scale of feature amplitude and in this study, delta band has the largest amplitude of all in Feature 2, suggesting that brain activity in the delta band is the highest.

## Discussion

This study evaluated the effect of sound therapy on nineteen cases suffering from chronic subjective tinnitus by recording their brain activity via measurement of EEG signal and by evaluating their respective RI through VAS using an innovative approach to extract relevant features from the wavelet decomposition. Eleven of the cases demonstrated RI in their respective VAS evaluation while eight subjects did not report any improvement in the level of loudness of their tinnitus. The underlying reasons of how RI occurs are not fully understood and are still under investigation (Roberts, 2007) but different therapies such as electrical stimulation have reported different effects on the subjects. Additionally, a recent study has reported that forward suppression of spontaneous firing in central auditory neurons with sound could be the cause of RI (Galazyuk et al., 2017). Furthermore, the occurrence of RI may depend on the correspondence of frequency spectrum of treatment and bandwidth of the tinnitus, loudness and duration of treatment and any negative thoughts, for example about tinnitus (Roberts et al., 2008; Dessai et al., 2014). Four features were used to evaluate the brain activity using the proposed 4-level wavelet decomposition and the mean value of delta band coefficients in channel FC3 were lower after the main treatment in cases with RI (*p* < 0.004). Our results indicate that tinnitus may cause additional brain activity in the delta band of the EEG and after an appropriate suppression through the broadband sound therapy, this activity may decrease. Our findings are in accordance with previous studies that used EEG and MEG mean power to compare the two groups (Kahlbrock and Weisz, 2008; Adjamian et al., 2012; Sedley et al., 2012; Meyer et al., 2014), and supports the notion that low frequency oscillations in brain activity of tinnitus patients may be triggered by the thalamocortical tinnitus input (Sedley et al., 2015).

Additionally, the brain map of the subjects showed that in the experiment, the activity in some regions of the brain, especially in frontal and temporal regions, may change after each stimulation suggesting that tinnitus and sound therapy may affect the brain activity in those regions.

Continuous mother wavelets such as Morlet, whose morphology has closer resemblance to EEG, may be better utilized to investigate the effect of tinnitus on the neuronal oscillations (Li et al., 2007). However, we used DWT in this study due to its preference to continuous wavelets, as explained in section 3.1. Also, to study the neuronal oscillations more thoroughly in tinnitus subjects, iterative methods can be implemented. Li et al. used Empirical Mode Decomposition (EMD) to extract brain waves in rats with epileptic seizures (Li et al., 2008). Their method is most suitable for offline calculations since its convergence could be time consuming (Wang and Zhou, 2013). In our proposed approach, iterative methods were not an immediate choice since it was important to decrease computation cost and as a result attempt to implement online analytical methods.

## Ethics Statement

This study was approved by the Ethics Committee of Iran University of Medical Sciences (IUMS) through Ethics Code of **IR.IUMS.REC1396.33176**. Furthermore participants provided written informed consent.

## Author Contributions

AA contributed to design of the work, data acquisition, analysis of data, and draft of the manuscript. MJ contributed to the concept and design of the work, supervising the analysis, and revising and finalizing the manuscript. SM contributed to the concept and design of the work, supervision of experiments, and revising the manuscript.

## Conflict of Interest Statement

The authors declare that the research was conducted in the absence of any commercial or financial relationships that could be construed as a potential conflict of interest.
